# A feasibility study using sodium alginate injection for penetrating abdominal trauma in a swine model

**DOI:** 10.1038/s41598-022-22186-0

**Published:** 2022-10-12

**Authors:** Daniel Barsky, Ami Ben Ya’acov, Linn Wagnert Avraham, Dean Nachman, Arik Eisenkraft, Yoav Mintz, Eyal Shteyer

**Affiliations:** 1 Faculty of Medicine, Hebrew University of Jerusalem, Ein Kerem, P.O. Box 12271, Jerusalem, Israel; 2 Juliet Keidan Pediatric Gastroenterology Institute, Shaare Zedek Medical Center and the Hebrew University of Jerusalem, Shmuel Bait St 12, Jerusalem, Israel; 3 Institute for Research in Military Medicine, the Hebrew University Faculty of Medicine, Ein Kerem, P.O. Box 12271, Jerusalem, Israel; 4 Department of Internal Medicine, Hadassah Medical Center, Ein Kerem, P.O. Box 91120, Jerusalem, Israel; 5 Department of General Surgery, Hadassah Hebrew University Medical Center, Ein Kerem, P.O. Box 91120, Jerusalem, Israel; 6 Faculty of Medicine, Institute for Research in Military Medicine, POB 12272, 91120 Jerusalem, Israel

**Keywords:** Diseases, Medical research, Drug development, Preclinical research

## Abstract

Penetrating abdominal injury is a major cause of death in trauma. Sodium alginate hydrogel, a hemostatic agent, offers a platform for targeting both mechanical and biological injuries. The current study assessed the effect of Very Low Viscosity (high) G (VLVG) alginate following abdominal trauma in a swine model of penetrating abdominal injury. Seven anesthetized pigs were instrumented with invasive monitoring catheters and abdominal trauma was introduced by laparoscopic hepatectomy. Ten minutes after the induction of hypovolemic shock, three animals were intra-abdominally administered with VLVG alginate (study group) and four animals with saline (control group). During 8 h of continuous monitoring, various hemodynamic and biochemical variables were measured and liver biopsies for histological evaluation were taken. Hemodynamically, VLVG alginate-treated animals were more stable than controls, as reflected by their lower heart rate and higher blood pressure (*p* < 0.05 for both). They also had lower levels of liver enzymes and lactate, and less histopathological damage. We show that VLVG alginate might be a promising new agent for reducing penetrating intra-abdominal injury, with hemostatic and biocompatibility efficiency, and tissue preserving properties. Future effort of integrating it with a dispersal device may turn it into a valuable pre-hospital emergency tool to improve survival of trauma casualties.

## Introduction

Penetrating abdominal injuries is common and carries significant morbidity and mortality in war zones and civilian settings^1–3^. Abdominal organs are especially vulnerable to penetrating trauma characterized by numerous injuries to solid organs, gastrointestinal tract, and vascular structures. Mortality from abdominal injuries occurs due to uncontrolled bleeding, organ damage, and prolonged evacuation^4^. Additionally, abdominal trauma may cause an inflammatory response that can aggravate tissue damage by causing diffuse bleeding, third space fluid loss, and pancreatitis. All of these may further cause complications such as abdominal compartment syndrome, acute coronary syndrome, and sepsis^4,5^. Various biomaterials for hemorrhage control have been developed for the military and civilian settings^6,7^. One of which is alginate, which is a safe, natural, biodegradable polysaccharide derived from brown algae. Due to its low toxicity, biocompatibility, and gel-forming properties, alginate is used for many indications, including hemorrhage control^8^. Alginate dressings maintain a physiologically moist environment that promotes tissue healing and regeneration. These non-adhesive dressings are highly absorbent and therefore are useful in treating different wound types^9,10^. In the presence of divalent cations such as calcium ions, alginate-based biomaterial forms a hydrogel, as the polymer becomes cross-linked. Therefore, alginate calcium dressings have become attractive in the biomedical field^11,12^. The use of alginate in penetrating abdominal trauma is not established yet. Taskin et al.^13^ have demonstrated the hemostatic properties of calcium alginate in an experimental rat model of splenic injury. They showed that the administration of calcium alginate gauze on an injured spleen reduced perioperative bleeding, as reflected by hematocrit values.

We have previously showed in several animal models the beneficial and protective effects of specific sodium alginate composition. Briefly, we found in a murine model of extended partial hepatectomy that calcium cross-linked alginate scaffolds on the liver remnant, significantly improved animal survival, reduced liver inflammation, and sustained hepatic synthetic function^14^. We further found in a drug-induced liver injury model that low molecular weight sodium alginate, also known as Very Low Viscosity (high) G alginate (VLVG) significantly reduced liver enzymes and pro-inflammatory cytokines^15^. In the current study, we tested the effect of VLVG alginate on hemodynamic stability and tissue injury following abdominal trauma in a swine model of penetrating abdominal injury.

## Results

Seven animals were included in the study, four in the control group receiving saline, and three in the study group treated with VLVG alginate. The following selected variables were compared between these 2 groups: heart rate, systolic blood pressure, (SBP), mean arterial pressure (MAP), creatinine, liver enzymes (ALT, AST), PH, base excess, lactate, fibrinogen and cortisol. These variables are presented in Table 1.

**Table 1:** Mean values (± SD) of variables recorded during 8 h of the experiment. Included are values at each time point between VLVG alginate and the control groups. Each panel represents the mean value of the measured parameter at that specific time point ± standard deviation.

	Time (hours)	1	2	3	4	5	6	7	8
Heart rate (BPM)	Alginate	73.33 ± 5.13	81.00 ± 7.00	83.33 ± 7.37	82.33 ± 7.50	82.33 ± 7.76	89.00 ± 11.35	94.00 ± 11.34	98.00 ± 18.87
	Control	91.00 ± 13.56	103.25 ± 7.41	113.25 ± 14.79	120.00 ± 21.83	121.75 ± 26.27	121.50 ± 29.69	116.00 ± 24.45	124.00 ± 25.10
SBP (mmHg)	Alginate	70.33 ± 12.22	73.67 ± 5.03	72.00 ± 1.00	69.67 ± 8.38	66.33 ± 5.68	66.33 ± 5.50	67.33 ± 7.37	67.67 ± 4.93
	Control	54.25 ± 12.06	56.25 ± 12.58	55.5 ± 14.27	56.00 ± 17.56	50.25 ± 8.46	51.25 ± 12.06	46.50 ± 10.10	46.25 ± 11.32
MAP (mmHg)	Alginate	49.33 ± 9.07	53.00 ± 3.60	51.00 ± 5.29	48.67 ± 9.01	44.00 ± 8.71	44.33 ± 6.80	44.00 ± 7.93	44.33 ± 7.57
	Control	39.00 ± 9.01	42.50 ± 9.29	42.75 ± 11.75	43.75 ± 14.99	37.75 ± 6.36	37.00 ± 7.74	34.00 ± 6.68	32.25 ± 8.26
Creatinine (µmol/L)	Alginate	136.33 ± 16.25	141.33 ± 20.84	144.33 ± 24.82	159.67 ± 37.07	179.00 ± 55.05	202.33 ± 62.14	214.67 ± 71.43	242.33 ± 86.31
	Control	177.75 ± 23.09	184.50 ± 37.00	194.00 ± 40.45	218.25 ± 55.82	242.75 ± 58.42	263.50 ± 60.83	283.50 ± 60.52	307.25 ± 61.64
AST (U/L)	Alginate	44.67 ± 15.94	42.67 ± 15.27	38.67 ± 14.04	38.67 ± 10.50	38.00 ± 9.64	37.00 ± 7.81	37.00 ± 5.29	38.00 ± 2.00
	Control	38.50 ± 5.44	40.25 ± 6.23	39.25 ± 4.19	41.50 ± 7.80	43.00 ± 11.80	50.75 ± 24.97	59.00 ± 46.15	80.00 ± 8.02
ALT (U/L)	Alginate	34.67 ± 9.29	33.33 ± 8.73	33.00 ± 9.16	34.00 ± 6.24	32.00 ± 8.18	31.33 ± 7.09	32.00 ± 7.55	31.68 ± 7.63
	Control	49.00 ± 11.77	48.75 ± 12.20	48.25 ± 12.84	49.00 ± 13.11	48.00 ± 12.35	49.00 ± 11.57	47.25 ± 10.01	47.25 ± 9.67
PH	Alginate	7.45 ± 0.06	7.48 ± 0.01	7.47 ± 0.01	7.45 ± 0.05	7.46 ± 0.08	7.49 ± 0.03	7.49 ± 0.04	7.47 ± 0.03
	Control	7.39 ± 0.04	7.43 ± 0.08	7.46 ± 0.05	7.4 ± 0.03	7.46 ± 0.04	7.47 ± 0.03	7.45 ± 0.05	7.45 ± 0.03
Base excess	Alginate	− 4.53 ± 3.49	− 6.13 ± 2.56	− 6.16 ± 3.43	− 6.63 ± 2.66	− 5.86 ± 2.37	− 6.1 ± 2.45	− 6.03 ± 2.04	− 5.767 ± 0.66
	Control	− 1.75 ± 0.73	− 1.82 ± 4.88	− 4.20 ± 2.28	− 3.00 ± 2.04	− 3.55 ± 3.83	− 4.65 ± 3.58	− 4.60 ± 3.16	− 4.22 ± 1.60
Lactate (mmol/L)	Alginate	2.87 ± 1.10	2.37 ± 0.69	1.76 ± 0.16	1.64 ± 0.13	1.63 ± 0.24	1.63 ± 0.44	2.04 ± 0.90	2.05 ± 0.72
	Control	4.45 ± 2.07	3.36 ± 0.80	3.19 ± 0.91	3.91 ± 1.58	4.12 ± 2.95	3.83 ± 2.33	2.98 ± 0.47	4.12 ± 1.5
Fibrinogen (mg/dL)	Alginate	450.00 ± 33.00	445.00 ± 32.00	445.00 ± 41.00	455.00 ± 42.00	462.00 ± 24.00	479.00 ± 23.00	490.00 ± 28.00	511.00 ± 43.00
	Control	369.00 ± 55.00	370.00 ± 49.00	374.00 ± 55.00	386.00 ± 44.00	386.00 ± 40.00	321.00 ± 128.00	413.00 ± 42.00	415.00 ± 42.00
Cortisol (nmol/L)	Alginate	312.00 ± 55.20	299.00 ± 63.58	318.00 ± 67.67	328.00 ± 137.57	338.00 ± 122.89	346.00 ± 137.56	333.00 ± 127.61	350.00 ± 150.47
	Control	347.00 ± 65.22	367.00 ± 5.47	376.00 ± 33.38	366.00 ± 41.73	388.00 ± 28.43	389.00 ± 45.89	402.00 ± 48.68	441.00 ± 76.96

### Hemodynamic measurements

**Figure 1:**
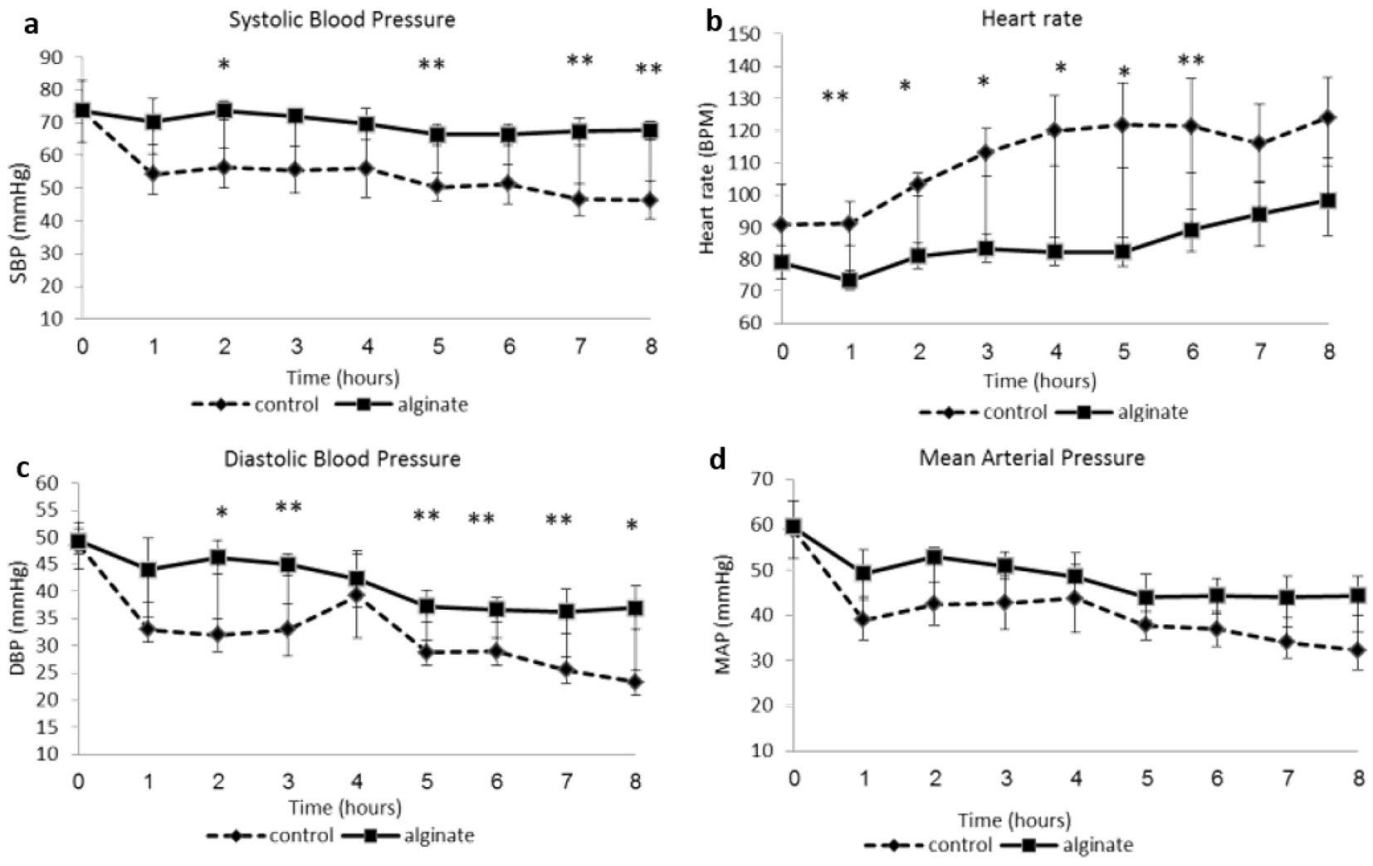
The effect of VLVG alginate on hemodynamic parameters. (**a**) Systolic blood pressure, (**b**) heart rate, (**c**) diastolic blood pressure, (**d**) mean arterial pressure. Data presented as means ± SD , n = 3 (alginate group), n = 4 (control group). *p < 0.05, **p = 0.05–0.1.

Compared with the control group, the VLVG alginate-treated group demonstrated significant changes in various hemodynamic measurements throughout the observation period. Specifically, systolic blood pressure (SBP), diastolic blood pressure (DBP), and mean arterial pressure (MAP) were significantly (*p* < 0.05) higher in the VLVG alginate-treated group compared with the control group, while heart rate (HR) was significantly (*p* < 0.05) decreased in the VLVG alginate-treated group compared to controls. These changes were and presented in Fig. 1.

**Figure 2:**
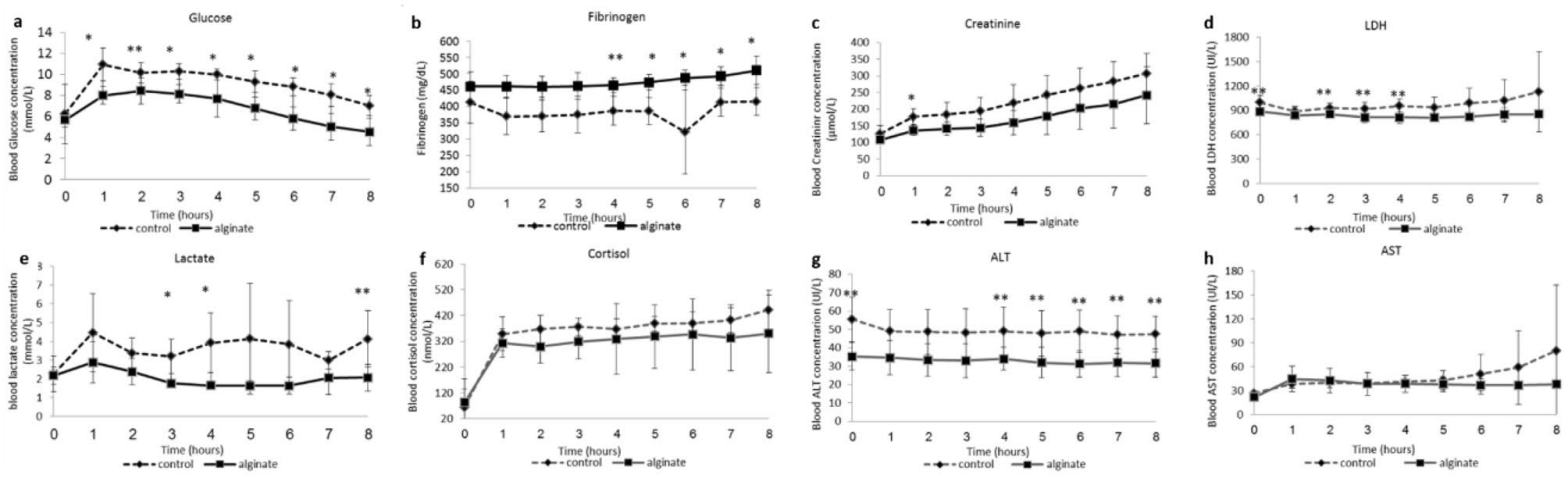
The effect of VLVG alginate on physiological parameters. (**a**) glucose, (**b**) fibrinogen, (**c**) Creatinine, (**d**) LDH, (**e**) Lactate, (**f**) Cortisol, (**g**) ALT, and (**h**) AST. Points are means ± SD deviation, n = 3 animals in the alginate group, n = 4 in the control group. *p < 0.05, **p = 0.05–0.1.

Postoperative laboratory values including glucose, fibrinogen, creatinine, lactate dehydrogenase (LDH), lactate, cortisol, and the liver enzymes alanine aminotransferase (ALT), aspartate aminotransferase (AST) that were recorded during the study are presented in Fig. 2. Levels of glucose and lactate were significantly lower in the VLVG alginate group (*p* < 0.05), while creatinine, LDH, and cortisol showed a similar trend but did not reach statistical significance. In addition, the mean levels of ALT and AST were higher in the control group compared with the VLVG alginate group, and the difference increased at the end of the observation period, though not reaching statistical significance. Fibrinogen levels were significantly higher in the VLVG alginate group compared with the control group.

### Blood counts

We also compared blood counts between VLVG-alginate-treated animals and controls. The levels of hemoglobin did not change significantly over time, neither did the levels of white blood cells (WBC), red blood cells (RBC), or platelets. Mean platelet level was higher in the VLVG group compared with the control group in all time points. No statistical differences could be observed concerning RBC and WBC over the observation period.

### Histopathology analysis

**Figure 3:**
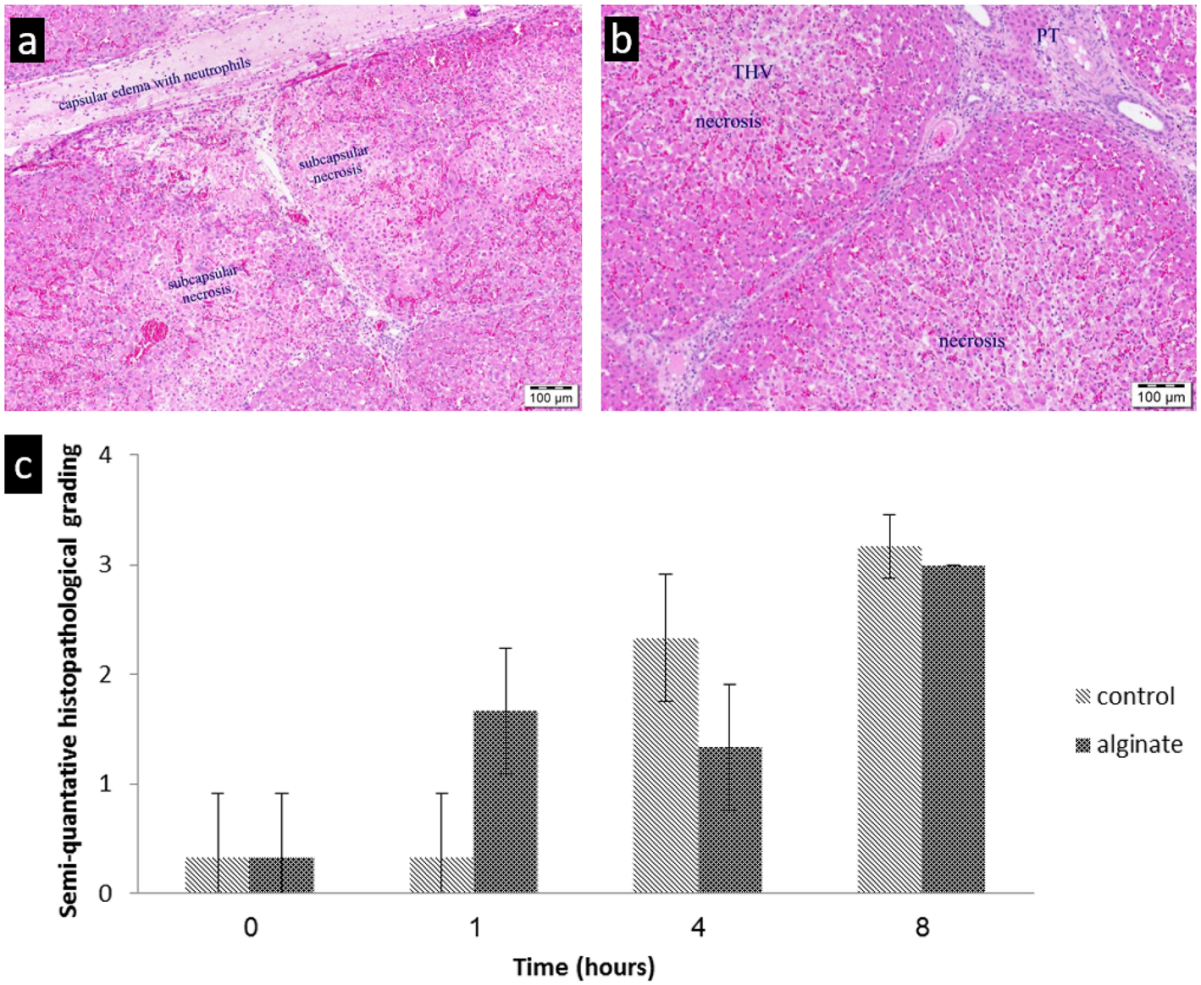
Histologic samples of liver biopsies 8 h after injury. (**a**) alginate-treated animal showing capsular edema and sub-capsular necrosis; (**b**) saline-treated animal showing hepatic lobular necrosis. PT- portal triad, THV- terminal hepatic venule. (**c**) Mean histopathological grading of the liver biopsies, comparing between VLVG alginate and the controls. Biopsies were taken at the beginning (t = 0), and after one hour, 4 h, and 8 h (at the end of the study) from injury. Histopathological changes were scored using semi-quantitative grading (0–4), according to change in severity: 0: No lesion- normal tissue; 1: minimal change; 2: mild change; 3: moderate change; 4: marked change. Each bar represents means ± standard error. n = 3 animals per group.

Liver biopsies were taken at time 0, and at one, 4, and 8 h after inducing the liver injury. Analysis of the biopsies demonstrated a pattern of acute neutrophil infiltration in liver septa, variable degrees of hepatic capsular edema, inflammation, and subcapsular necrosis (Fig. 3a,b). There were also areas of central lobular hemorrhage and necrosis in biopsies from both groups. Notably, in liver samples taken later during the study, we found a lesser degree of histopathological damage in the alginate-treated animals when compared with liver samples from the control group, as reflected by semi-quantitative grading (Fig. 3c).

## Discussion

Penetrating abdominal trauma is often seen in gunshot or stab wounds. As the liver occupies a large portion of the abdominal cavity, up to 30% of penetrating abdominal trauma results in liver damage^16^. Other organs that are commonly involved are the small and large intestines, as well as vascular structures^16–18^. Because most of deaths take place before reaching any setting with definite care, e.g., the field hospital, effective hemostatic techniques are highly valuable. Short-term mortality from penetrating abdominal trauma is caused by hemorrhage, and long-term mortality is usually due to sepsis and multi-organ failure^19–22^. In the current study we demonstrated that injecting VLVG alginate into the abdominal cavity of swine after inducing a controlled abdominal injury kept the animals hemodynamically stable and reduced the degree of organ damage.

In order to establish a valid trauma model, we demonstrated changes in several vital components that reflect the degree of organ damage and shock. These include hemodynamic parameters, laboratory indices, such as blood and biochemical profile, endocrine profile, and markers of hepatic tissue damage^23–26^. Also, liver tissue samples in the setting of shock could demonstrate certain histopathological changes such as central lobular necrosis^27^, related to the level of injury. Our model demonstrated changes in all of these components among the control group, thus showing it is an appropriate model for trauma, hemorrhagic shock, and liver injury. Although hemorrhagic shock was induced, the degree of damage was not sufficient to inflict mortality, and therefore we could not conclude from this study whether VLVG alginate improved survival. Hemodynamic monitoring revealed lower HR and higher blood pressure values (SBP, DBP, and MAP) in the VLVG alginate–treated animals. Since hemodynamic parameters are used to assess the degree of hemorrhagic shock, our results suggest that animals treated with VLVG alginate were in a lower level of shock.

In previous studies in murine models, we showed the beneficial effect of VLVG alginate^14,15^. These studies demonstrated that the effect of this specific low molecular weight alginate is not merely physical, and it shows superior effects over other alginate formulations due to different biochemical properties, leading to better absorption through membranes. These findings led to the current study in which we examined whether VLVG alginate could have a protective effect on abdominal organs, especially the liver. During stress, cortisol levels rise leading to an increase in blood glucose levels due to glycogenolysis, gluconeogenesis, and insulin resistance^28^. In the current study, as depicted in Fig. 2f, in all time points cortisol levels were lower in the treated group, yet it did not reach statistical significance. This could imply that the VLVG treated group was in lower stress, but it will have to be further elucidated in a larger group of animals. Another shock marker is lactate. Lactate is formed in anaerobic metabolism when tissues lack oxygen due to circulatory failure. As such, it is a marker of tissue hypoperfusion and shock^29^. Indeed, in the current study, the mean blood levels of lactate showed variability. They were reduced in the VLVG-treated group throughout the experiment (Fig. 2e) but significant difference was observed only at the 3-, 4- and 8-h time-points, suggestive of better tissue perfusion. Fibrinogen, a marker of hemostasis, is a substrate for clot formation and is known to be low following trauma with massive hemorrhage due to consumption. Furthermore, lower levels of fibrinogen after hemorrhage are associated with worse outcomes^30,31^. In the current study, VLVG-treated animals constantly showed higher levels of fibrinogen, suggesting less activation of the coagulation cascade, strengthening the notion that alginate has hemostatic properties.

Trauma is a multi-organ condition that might cause damage to distinct organs such as the kidneys. Acute renal failure is a common feature in major trauma and caused by renal hypoperfusion or late consequences such as rhabdomyolysis or abdominal compartment syndrome. Acute renal failure is represented by serum creatinine concentration^32,33^. In the current study, creatinine levels were lower in the VLVG-treated group throughout the experiment (Fig. 2c) but significant difference was observed only in the first hour.

Blood levels of liver enzymes are known to correlate with the degree of liver damage in various clinical conditions, including trauma^34,35^. In the current study, levels of ALT were higher in the control group throughout the experiment with significant difference from the 4th hour to the end of the monitoring period, suggesting a positive effect of VLGL in reducing the extent of liver injury. However, it is important to note that baseline ALT level was higher in the control group. Unlike ALT, AST was higher in the control group only from the 6-h time point but the difference between groups did not reach statistical significance. Lastly, LDH, a non-specific marker for organ injury, was higher throughout the experiment (Fig. 2d) in the control group but reached statistical significance at the 2-, 3-, and 4-h time points. The exact mechanism of action in which VLVG sodium alginate affect the liver is still unknown, but previous studies in mice suggest a preserving effect on the liver tissue^15^. In this study the histopathological analysis showed that the VLVG-treated group had a lesser degree of damage in the middle and final hours of the study, further strengthening the hypothesis of hepatic preservation properties by VLVG.

Our study has several limitations. Only 7 animals were included in the study which did not allow sufficient statistical power. Consequently, we used higher cutoffs for p-values and comparison of a tendency to demonstrate the effect of alginate. As the current research was a feasibility study, we used only female animals to minimize confounders of sex and to gain a favorable surviving profile. Although the same degree of liver injury was induced in all animals, it was impossible to compare or measure the amount of intra-peritoneal blood loss since saline and alginate were administered to the intra-peritoneal cavity and influenced the blood volume in a way that does not allow its measurement. In addition, due to ethical limitations, the length of observation in our study was rather short, not allowing to fully assess survival and long-term effects of VLVG, although it should be noticed that there is evidence that sodium alginate penetrates membranes and works through systemic absorption, and it has a positive effect on the peritoneum as it reduces the extent of intra-peritoneal adhesions^15,36^. Future studies would allow substantiating these findings. Moreover, we have shown that our animal model can help with the development and testing of novel hemostatic agents for the treatment of intra-abdominal bleeding in the field, whether it is a civilian or a military setting.

In conclusion, this small, proof-of-concept study showed that treatment with alginate, specifically VLVG, improved the hemodynamic, metabolic, and liver histopathological parameters following penetrating abdominal trauma in pigs, when compared with untreated animals. Further studies are warranted to corroborate these findings.

## Methods

### Animals

Female domestic swine (45–50 kg) were maintained in an AAALAC accredited facility of the Hebrew University Faculty of Medicine, Jerusalem, Israel. All animals were allowed to acclimate for three days and examined by a veterinarian to ensure good health before starting the surgery. Food was withheld the night before the experimental procedure, but free access to drinking water was provided. The joint ethics committee of the Hebrew University Faculty of Medicine and the Hadassah Medical Center approved the study (approval number MD16148533). The study adhered with the ARRIVE guidelines. All methods were performed in accordance with the relevant guidelines and regulations. Animals were randomly assigned to the two experimental groups.

### Instrumentation and monitoring

Animals were sedated with intramuscular (IM) Xylazine (1 mg/Kg, IM, Eurovet Animal Health B.V. Netherlands) and Ketamine (10 mg/Kg, IM, Vétoquinol SA France) before induction. Next, intravenous (IV) anesthesia was initiated by cannulating the ear vein, and animals were placed in a supine position. This was followed by administering a mixture of Tramadol (5 mg/Kg, IM, Rafa Laboratories Ltd. Israel), Diazepam (2 mg, IV, TEVA Pharmaceutical Industries Ltd. Israel), Ketamine (400 mg, IV), and Propofol (1–4 mg/Kg, IV, Fresenius Kabi Austria GmbH Austria), with prophylactic use of Cefazolin (1 g, IV, Panpharma S.A. France). Animals were then intubated with a cuffed elastic endotracheal tube (7.0 mm, Portex Tracheal Tube). Animals were ventilated using controlled mechanical ventilation (Datex Ohmeda SmartVent 7900, Datex-Ohmeda Inc, Madison WI USA/Narkomed 2B Anesthesia Machine—North American Drager), with anesthesia maintained using 2% isoflurane (Piramal Critical Care Inc. PA, USA) in 100% oxygen. Tidal volume was set to 10 mL/kg with a respiratory rate of 13–15 breaths per minute and inspiratory:expiratory ratio of 1:2 adjusted to reach an end-tidal PCO_2_ of 35 mmHg at baseline. Vital signs (blood pressure, heart rate, respiratory rate, and temperature) were recorded every 5 min. Moreover, 15 ml blood samples for each time point were withdrawn for a serum test set, which included a complete blood count (CBC), biochemical variables, blood gas analysis, and metabolic profile, using an arterial line inserted through the left common carotid artery.

### Experimental procedure

**Figure 4:**
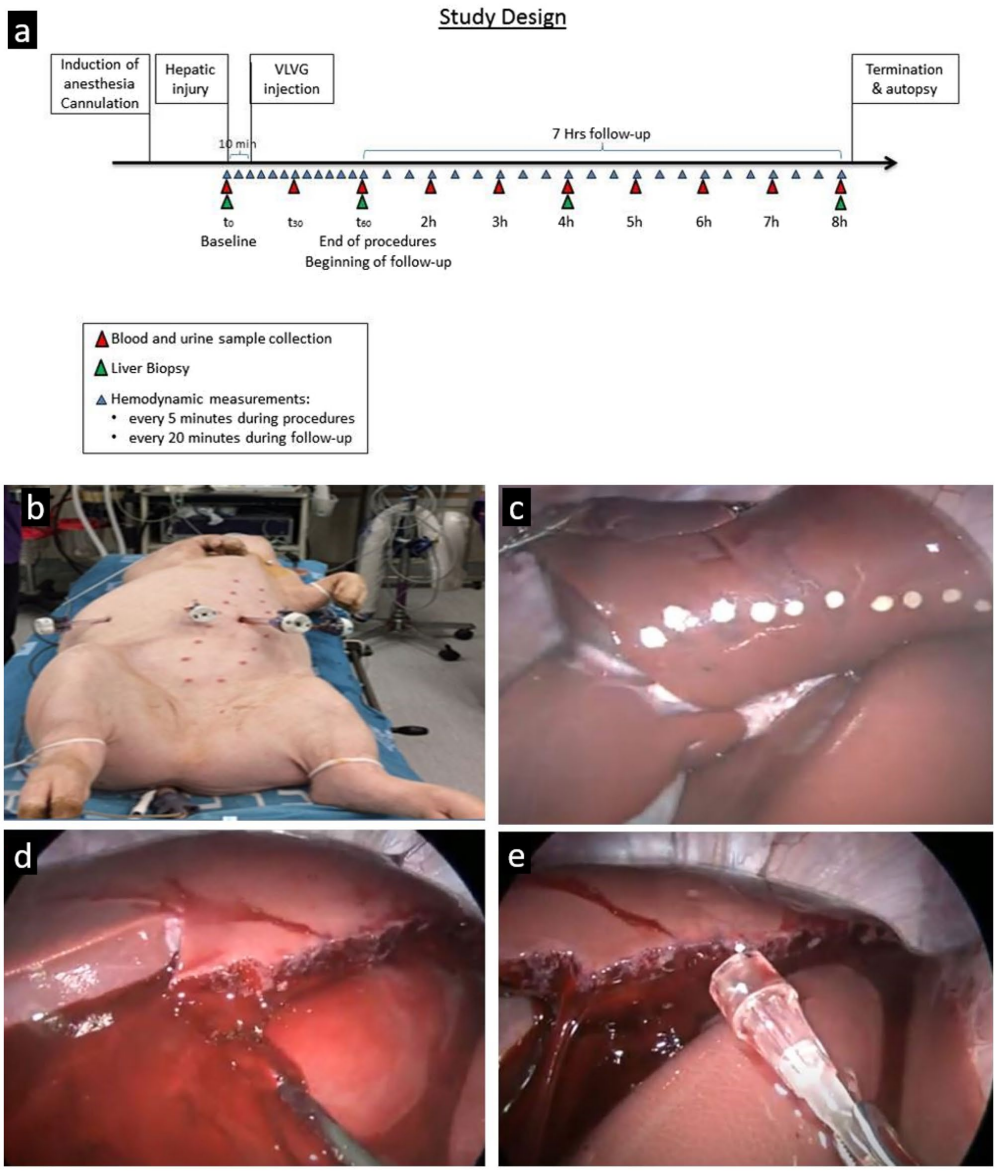
Study design and induction of liver injury. (**a**) a scheme of the study design; (**b**) pneumoperitoneum was induced using a Veress needle, and three trocars were introduced to perform laparoscopy; (**c-e**) marking the edge of the liver lobe, 4 cm from the portal vein, followed by a left lobectomy.

A scheme of the study design is presented in Fig. 4a. After the animals were stabilized, pneumoperitoneum was induced using a Veress needle to a pressure of 12 mmHg, and three trocars were introduced to perform laparoscopy (Fig. 4b). During this procedure, a small biopsy was taken from the left lobe of the liver. Then a left lobectomy was performed throughout a plain at a constant distance of 4 cm from the portal vein (Fig. 4c–e). Ten minutes later a catheter was introduced via the laparoscopic trocar and the treatment was induced to the abdominal cavity in the following order:

Control animals were administered 200 ml saline on the liver remnant and 100 ml were administered on the peritoneum and on the small and large intestines. VLVG-treated animals were administered 300 ml of 2% of VLVG which was administered similarly to control animals. All animals were continuously monitored for 8 h, during which hemodynamic parameters were recorded and blood samples were withdrawn. Liver biopsies were taken at 0-, 4-, and 8-h time points and were analyzed by a veterinary pathologist. Histopathological changes were scored using semi-quantitative grading (0–4) according to change in severity from normal morphology. After 8 h the animals were sacrificed using an injection of potassium-chloride solution.

### Alginate

To obtain a free-flowing form of low molecular weight sodium alginate (Very Low Viscosity (high) G alginate, VLVG), we prepared a 2% solution by dissolving 6gr of VLVG (NovaMatrix, FMC biopolymers, Drammen, Norway) in 300 ml of saline at room temperature for over-night. The alginate solution was freshly made and appeared clear.

### Statistical analysis

Statistical analysis was conducted using IBM SPSS Statistics for Windows, version 25 (IBM Corp., Armonk, N.Y., USA). Descriptive statistics of the variables included mean, median, range, and standard deviation. To compare the study group with the control group at a specific time point, the non-parametric Mann–Whitney test was used. This test uses multiple comparisons; thus, the Bonferroni correction of the significance level was used, and a *p* value <  = 0.005 was considered statistically significant. To assess if there was a trend over time, we used Friedman’s non-parametric test. In order to simultaneously assess the effect of treatment (i.e., normal saline vs VLVG alginate), time, and the interaction between them, we used the repeated measures ANOVA model, with the Greenhouse-Geiser test for assessing time effect. Non-parametric tests were applied to specific comparisons, due to the small sample size. However, when data was analyzed for all animals, for all time points (repeated measures ANOVA), a matrix of 63 measurements was included in the analysis, thus creating a robust data base (concerning the normal distribution assumption), and enabling the application of the above ANOVA model.

All tests were 2-tailed, and *p* <  = 0.05 was considered as statistically significant. However, due to the relatively small sample, a *p* value between 0.05 and 0.1 was determined as borderline significant.

## Data Availability

The datasets used and/or analyzed during the current study are available from the corresponding author on reasonable request.
